# Primary Human Airway Epithelial Cell-Dependent Inhibition of Human Lung Mast Cell Degranulation

**DOI:** 10.1371/journal.pone.0043545

**Published:** 2012-08-27

**Authors:** Neil Martin, Andrew Ruddick, Greer K. Arthur, Heidi Wan, Lucy Woodman, Christopher E. Brightling, Don J. L. Jones, Ian D. Pavord, Peter Bradding

**Affiliations:** 1 Department of Infection, Immunity and Inflammation, Institute for Lung Health, University of Leicester, United Kingdom; 2 Cancer Studies and Molecular Medicine, RKCSB, University of Leicester, Leicester, United Kingdom; University of Rochester Medical Center, United States of America

## Abstract

**Introduction:**

Chronic mast cell activation is a characteristic feature of asthma. BEAS-2B human airway epithelial cells (AEC) profoundly inhibit both constitutive and IgE-dependent human lung mast cell (HLMC) histamine release. The aim of this study was to examine the regulation of HLMC degranulation by primary AEC from healthy and asthmatic subjects, and investigate further the inhibitory mechanism.

**Methods:**

HLMC were co-cultured with both BEAS-2B and primary AEC grown as monolayers or air-liquid interface (ALI) cultures.

**Results:**

Both constitutive and IgE-dependent HLMC histamine release were attenuated by BEAS-2B, primary AEC monolayers and ALI cultures. This occurred in the absence of HLMC-AEC contact indicating the presence of a soluble factor. Unlike healthy ALI AEC, asthmatic ALI-AEC did not significantly reduce constitutive histamine release. AEC inhibitory activity was transferable in primary AEC monolayer supernatant, but less active than with Transwell co-culture, suggesting that the inhibitory factor was labile. The AEC inhibitory effects were attenuated by both AEC wounding and pertussis toxin, indicating the involvement of a G_0_/G_i_ receptor coupled mechanism. Solid phase extraction of lipids (<10 kDa) removed the AEC inhibitory activity. The lipid derivatives resolvin D1 and D2 and lipoxin A_4_ attenuated HLMC histamine release in a dose-dependent fashion but were not detectable in co-culture supernatants.

**Conclusions:**

Primary AEC suppress HLMC constitutive and IgE-dependent histamine secretion through the release of a soluble, labile lipid mediator(s) that signals through the G_0_/G_i_ receptor coupled mechanism. Manipulation of this interaction may have a significant therapeutic role in asthma.

## Introduction

Chronic mast cell activation is a characteristic feature of asthma [1]; [2]. There is ongoing production and release of mast cell-derived autacoid mediators and cytokines [3] and morphological evidence of degranulation within asthmatic airways [4] Mast cells infiltrate three key structures in asthma: the airway epithelium [5], the airway submucosal glands [6], and the airway smooth muscle [7]. Recent work has highlighted important bi-directional interactions between human lung mast cells (HLMC) and airway smooth muscle, including the ability of ASM to increase constitutive mast cell degranulation [8]; [9]. These interactions are likely to promote ASM dysfunction in asthma.

The outcome of mast cells interacting with the airway epithelium is poorly understood. Airway epithelial cells (AEC) are capable of suppressing mast cell chymase expression [10], and supporting mast cell survival [11], in part through the generation of the essential mast cell growth factor, stem cell factor. AEC activated with various stimuli produce TSLP which may induce IL-13 release from cultured mast cells derived from peripheral blood progenitors [12], and mast cells are required for epithelial TSLP expression in a model of allergic rhinitis [13].

We have previously demonstrated that HLMC in contact with BEAS-2B AEC exhibit a marked reduction in both constitutive and IgE-dependent HLMC histamine release [14]. Since the airway epithelium in asthma is denuded and expresses an inflammatory phenotype with impaired repair responses [15], we proposed the following hypothesis: that the role of the healthy intact epithelium is to keep mast cells in a quiescent state, and that tissue insults such as those caused by infection or that present in asthma lead to epithelial damage and denudation which consequently leads to the loss of this bronchoprotective function. If true, this may be critically important in the development of airways hyperreactivity, variable airflow obstruction and airway remodelling.

To further our understanding of the mechanisms regulating HLMC function by AEC, we have now studied the effects of primary human AEC including air liquid interface (ALI) cultures, derived from both healthy and asthmatic subject cultures, on HLMC degranulation.

## Methods

### BEAS-2B Cell Culture

The BEAS-2B epithelial cell line was purchased from the European Collection of Animal Cell Cultures (Porton Down, Wiltshire, UK). Cells (passages 8–12) were grown on human plasma fibronectin-coated T75 culture flasks in BEBM media (Clonetics Cat. No. CC4175), with an added enhancement bullet kit (Clonetics Cat. No. CC4175), Pen/Strep (5 ml) and fungizone (5 ml) to create basal epithelial growth media (BEGM). BEAS-2B were then passaged on to human plasma fibronectin-coated 16-well 0.40 µm Transwell plates and then grown to confluence prior to use in assays.

### HLMC Purification and Culture

All subjects donating lung tissue gave written informed consent, and the study was approved by the Leicestershire Research Ethics Committee. HLMC were dispersed from macroscopically normal lung obtained within 1 h of resection for lung cancer using immunoaffinity magnetic selection as described previously [14]. Final mast cell purity was >99%, and viability >99%. HLMC were cultured in DMEM, 10% FCS, antibiotic/antimycotic solution, SCF 100 ng/ml, IL-6 50 ng/ml and IL-10 10 ng/ml [14].

### Air-Liquid Interface Cultures

Asthmatic subjects (n = 6) and healthy controls (n = 6) were recruited from Glenfield Hospital, Leicester, UK. Asthmatic subjects had a consistent history and objective evidence of asthma, as described previously [7] a summary of their main clinical characteristics is given in Table 1. Subjects underwent extensive clinical characterization including video-assisted fiberoptic bronchoscopic examination. The study was approved by the Leicestershire Ethics Committees. All patients gave their written informed consent. Primary epithelial cells were isolated from bronchial brushes, grown to confluence on 1% PureCol-coated surfaces (Inamed Biomaterials, Nutacon, The Netherlands) as submerged cultures using bronchial epithelial growth medium (BEGM, Lonza Verviers, Belgium) supplemented with 0.3% Fungizone® antimycotic (Gibco, Invitrogen, Paisley, UK) and 1% antibiotic-Antimycotic (AA) (Gibco). When confluent, epithelial basal cells were seeded into PureCol-coated Transwell® inserts (12 mm diameter, 14 µm polyester membrane) (Corning, Lowell, MA), firstly in submerged culture to confluence, secondly in Air-Liquid-Interface (ALI) using ALI medium (50∶50 BEGM:DMEM [Gibco], supplemented with 0.3% Fungizone®, 1% AA and 100 nM retinoic acid [Sigma, Poole, UK]). Ciliated cultures with high ciliogenesis were used for experiments.

**Table 1:** The clinical characteristics of the ALI culture donors. All had a clinical, symptom based diagnosis of normal or asthma.

Asthmatic	n1	n2	n3	n4	n5	n6
Sex	Male	Male	Male	Female	Female	Female
Age (yrs)	20	62	52	57	42	36
ICS Dose	0	2000	0	0	200	0
FEV1/FVC (%)	85	63				87
% pred FEV1	100	86	87	103	114	115
FEV1 postbronchodilator	4.20	3.40	3.51	2.91		3.15
PC20methacholine	2.10			0.03	0.03	>16
Atopy(+2spts)	Yes	No	No	Yes	Yes	No
IgE	357	266	25	Not done	91	Not done
Bloodeosinophils	0.70	1.60	0.31	0.47	0.14	0.08
SputumEosniophils	10	27		7.00	2.80	Not done
**Non-Asthmatic**	**n1**	**n2**	**n3**	**n4**	**n5**	**n6**
Sex	Male	Male	Male	Male	Female	Female
Age (yrs)	70	62	23	24	38	37
ICS Dose	0	0	0	0	0	0
FEV1/FVC (%)	79	81	82	79	85	76
% pred FEV1	106	94	110	89	105	110
FEV1 postbronchodilator	3.00	3.4	5.15		4.2	3.7
PC20	>16	>16	>16	>16	>16	>16
Atopy(+2spts)	No	Yes	Yes	No	No	No
IgE	13.8	41	Not done	Not done	Not done	Not done
Eos (per bld)	0.04	0.13	0.17	0.05	0.06	0.14
SputumEosinophils		0.25		Not done	0.25	0.25

### Co-culture Experiments

#### HLMC-BEAS-2B co-culture

Three conditions were compared initially: i) HLMC (5×10^4^ **)** in direct contact with confluent BEAS-2B cells cultured in 24 well plates, ii) BEAS-2B grown to confluence initially on an inverted 0.4 µm Transwell membrane which was then inserted into the plate in the correct orientation, with HLMC (5×10^4^) then added to the top chamber, and iii) HLMC (5×10^4^) cultured alone on plastic in the bottom of a 24-well plate. The cells were cultured for 16 h, the media then removed gently, any non adherent cells in the removed media recovered by centrifugation and the cells returned to their respective wells in fresh media. They were then activated with anti-human IgE for 30 minutes, supernatants removed and the cells recovered by centrifugation. The remaining cells were then lysed with sterile water to allow for measurement of cell histamine content.

In further experiments, BEAS-2B were grown to confluence in the top of a Transwell membrane, and HLMC added to the bottom chamber.

#### HLMC-ALI AEC co-culture

These were carried out exactly as the latter BEAS-2B experiments with ALI AEC cultures grown to confluence as described above and then transferred into a fresh well with HLMC added to the bottom chamber.

### Mast Cell Activation

Mast cells were sensitised using 2.5 µg/ml human IgE (Calbiochem, Darmstadt, Germany) for at least 1 h, washed and re-suspended in fresh media. They were stimulated with anti-IgE (Mouse IgG Hybridoma Reagents, Baltimore, USA) at a final concentration of 1/1000 for 30 minutes. Pertussis toxin (PTX), (Sigma, UK) was used at a final concentration of 500 ng/ml, the EP_2_ receptor antagonist AH6809 (Sigma, UK) was used at a final concentration of 10 µM based on our previous experience with this [15]. AEC wounding was carried out by placing a simple X on the cultured cells with a 10 µl pipette tip.

### Mediator Assays

Histamine was measured by sensitive radioenzymatic assay as described previously [14]. Lipoxin A_4_ was measured by ELISA (Oxford Biomedical Research, Oxford, MI, USA) according to the manufacturer’s instructions.

### Fractionation of AEC Supernatants and Co-culture Supernatants

We fractionated AEC supernatants from primary AEC monolayers using reversed phase C^18^ solid phase extraction (SPE)(Phenomenex Strata-X, 33 µm, 85 A°, <10 kDa) (Phenomenex, Torrance, CA, USA) to remove small lipid mediators [16]. The cartridge was conditioned with methanol followed by water prior to the application of the supernatant. The lipid-depleted AEC media was collected then incubated with HLMC and the inhibitory effect on constitutive histamine release was investigated.

### Measurement of Resolving Mediators Using Liquid Chromatography (LC)-mass Spectrometry

We used liquid chromatography (LC)-mass spectrometry (MS) to look for the presence of resolving mediators of inflammation (lipoxins, resolvins, protectins, maresins) in both primary AEC monoculture supernatants and HLMC-co-culture supernatants. For targeted determination, cell culture media were subject to C^18^ solid phase extraction (see above), which purifies lipid mediators and removes interfering substances. The extracted samples were kept as solutions in methanol at −20°C; these were then dried by rotary evaporation and reconstituted in LC solvent prior to analysis. Samples were analysed using a Waters (Manchester, UK) Acquity ultra performance (UP)LC system coupled to a Waters Xevo TQ triple quadrupole mass spectrometer operated in negative ion mode with selected reaction monitoring. *Chromatography*: Solvents were 0.02% acetic acid (solvent A) and 45% acetonitrile (solvent B), used in a gradient system varying between 50∶50 A:B and 20∶80 A:B. The column was a Waters Acquity BEH C_18_ 1.7 µm, 2.1×150 mm. Injection volume was 10 µl, total run time 17 minutes. *Mass spectrometry*: Capillary voltage was 2.6 kV, cone voltage 25 V; desolvation temperature was 550°C with gas flow 800 l/hr. Collision gas flow was 0.2 l/min. Ion transitions monitored for selected reaction monitoring were: resolvin D1 and D2 375.2 → 141.2; lipoxin A_4_ 351.2 → 115.2; deuterated-lipoxin A_4_ 356.2 → 115.2; resolvin E1 349 → 195; resolvin E2 333.2 → 199.2; protectin D1 359.2 → 206.2; maresin 1 359.2 → 250.2 [17]; [18]. Deuterated internal standard (d5-lipoxin A_4_) was added to the media, enabling the accurate quantitative determination of each mediator.

### Statistics

All statistical analysis was carried out using SPSS Version 14.1 (IBM). All experiments were performed with at least 4 replicates per condition. The mean of the replicates was used for statistical analysis. Comparisons across groups were made using a repeated measures ANOVA with post test Bonferroni correction, a p value of <0.05 was considered statistically significant.

## Results

### A Soluble Factor(s) Mediates BEAS-2B-dependent Inhibition of Constitutive and IgE-dependent HLMC Degranulation

**Figure 1:**
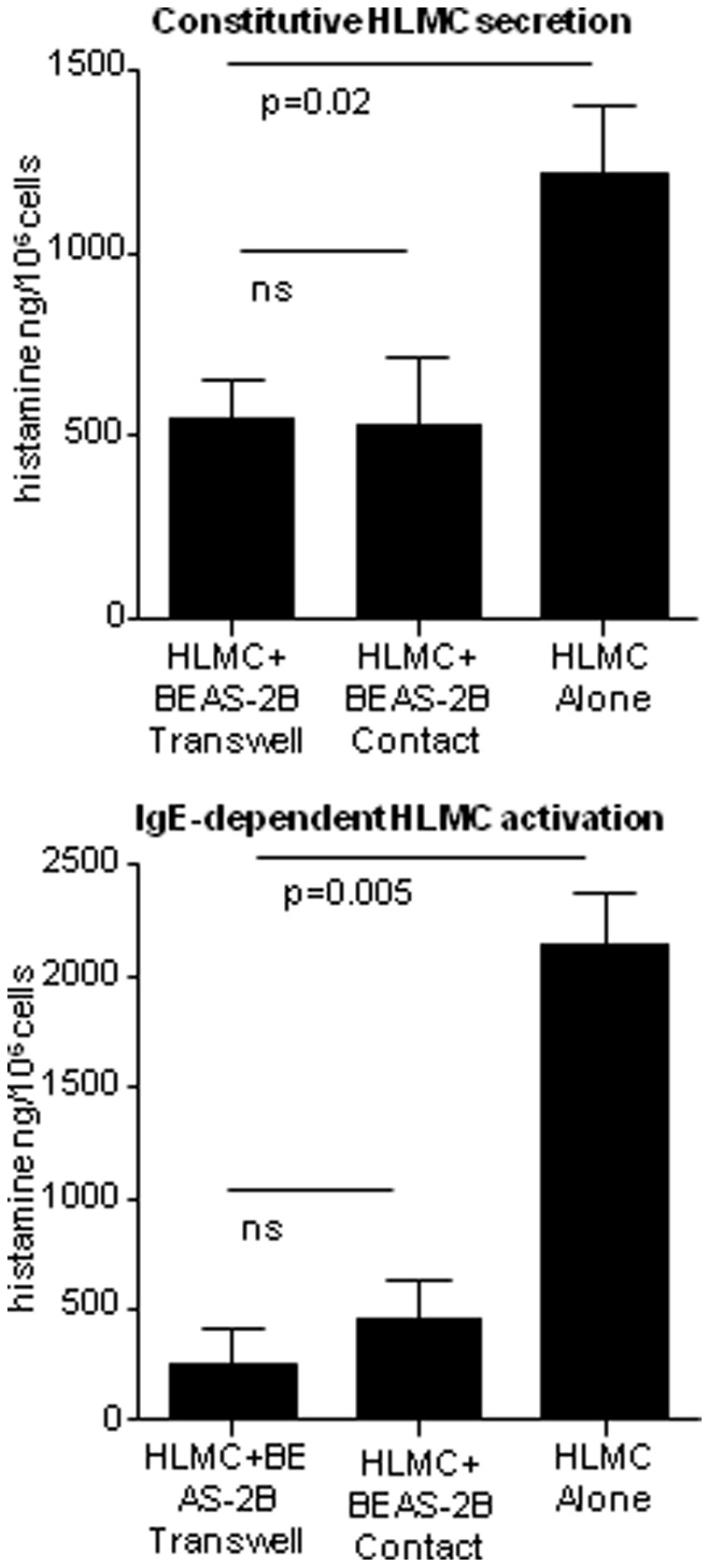
Constitutive (16 h) and IgE-dependent (30 min) HLMC histamine secretion in monoculture and in the presence of BEAS-2B cells, either in contact or on either side of a Transwell membrane (BEAS-2B on the underside of the membrane, HLMC on the upperside of the membrane). Mean ± SEM ng/10^6^ cells (N = 3 HLMC donors).

**Figure 2:**
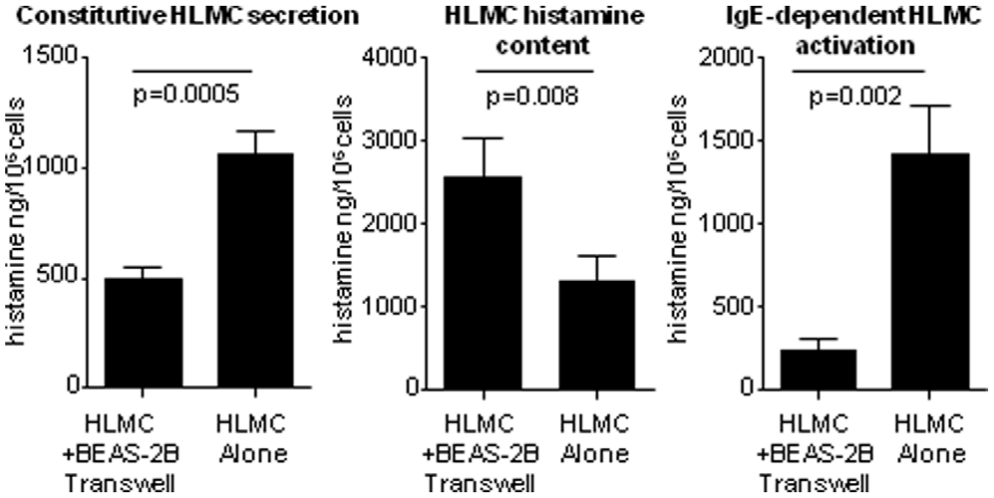
Constitutive (16 h) and IgE-dependent (30 min) HLMC histamine secretion in monoculture or in the presence of BEAS-2B cells separated by a Transwell membrane (HLMC in the bottom chamber, BEAS-2B in the top chamber). Retained cell histamine content prior to activation is also shown. n = 7 HLMC donors. Mean ± SEM ng/10^6^ cells.

Initially we extended our original work examining the suppression of HLMC degranulation in direct contact with confluent BEAS-2B cells, by comparing the co-culture of HLMC and BEAS-2B cells separated by a 0.40 µm Transwell membrane (BEAS-2B on the underside, HLMC in the top chamber). Analysis of cell supernatants revealed a 55% (95% CI: 35%–76%; p = 0.002) suppression of constitutive histamine release over 16 h in the presence of BEAS-2B in either direct contact (56%; 95% CI: 35%, 76%; p = 0.002) or separated by a Transwell membrane (54%; 95% CI: 50%, 85%; p = 0.002) when compared to HLMC cultured alone (Fig.1). There was also a pronounced suppression of IgE-dependent histamine release in the presence of BEAS-2B cells (79%, 95% CI: 62%, 92%; p = 0.005), and in the presence of the Transwell membrane (89%, 95% CI: 66%, 99%; p = 0.005) (Fig.1). Similar results were seen when BEAS-2B were present on the top of a Transwell membrane with HLMC in the bottom chamber (n = 7 HLMC donors)(Fig.2). This was associated with an increase in cellular histamine content in HLMC co-cultured with BEAS-2B Transwells compared to HLMC cultured alone (n = 7) (Fig.2).

BEAS-2B airway epithelial cells therefore consistently inhibit HLMC constitutive and IgE-dependent histamine release, but cell-cell contact is not required indicating that a soluble mediator(s) is involved.

### Air Liquid Interface Cultures of Primary Airway Epithelial Cells Inhibit HLMC Degranulation

**Figure 3:**
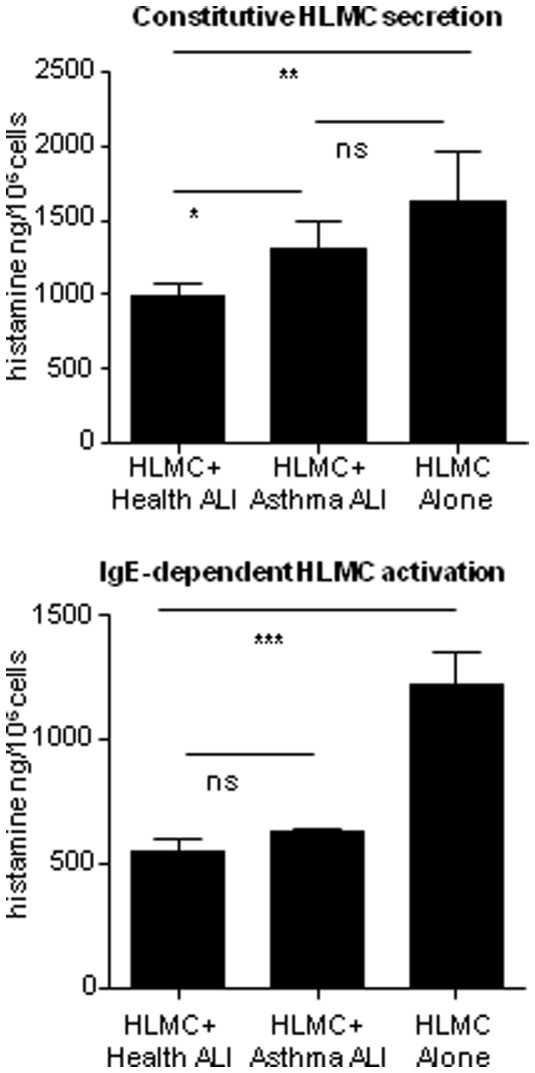
The effects of HLMC co-culture with healthy and asthmatic ALI AEC on constitutive (16 h) and IgE-dependent (30 min) HLMC histamine secretion compared to HLMC cultured alone, n = 6 HLMC donors. Mean ± SEM ng/10^6^ cells. (p<0.05*, p<0.01**, p<0.001***, ns = not significant).

We then repeated the latter Transwell experiments described above using ALI AEC cultures that had been grown from well characterised donors either with or without a diagnosis of asthma. Constitutive (16 h) histamine release was significantly lower from HLMC co-cultured with healthy ALI AEC compared to HLMC co-cultured with asthmatic ALI AEC (p = 0.03) or HLMC cultured alone (p = 0.001) (n = 6 healthy AEC donors, 6 asthmatic AEC donors and 6 HLMC donors)(Fig.3). Asthmatic ALI AEC did not significantly reduce constitutive histamine release compared to HLMC cultured alone (p = 0.07) (Fig.3). The presence of ALI AEC also significantly suppressed IgE-dependent histamine secretion compared to HLMC monoculture (p<0.0001) and healthy and asthmatic ALI AEC were equally effective in this respect.

### AEC Inhibitory Activity is Transferable in Primary AEC Monolayer Supernatant

**Figure 4:**
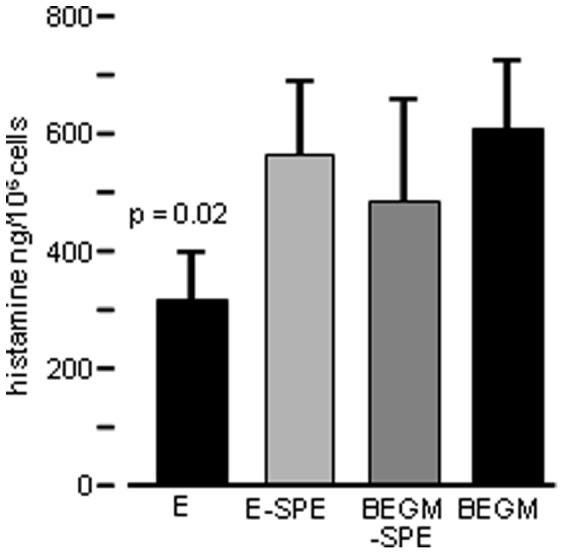
HLMC histamine release in different culture media. E – primary AEC monolayer supernatant; E-SPE – SPE-processed (lipid-deplete) AEC supernatant; BEGM-SPE – SPE-processed BEGM media; BEGM – unprocessed media. Mean ± SEM of 4 experiments using 2 AEC donors and 2 HLMC donors. HLMCs cultured in AEC supernatant showed suppressed histamine release (p<0.05) whereas there is no significant suppression in lipid-deplete supernatant.

To assess further, whether the AEC-inhibitory activity is transferable, we measured constitutive histamine release from HLMC cultured in primary AEC monolayer supernatants for 16 h, compared to control AEC media. There was a significant reduction in constitutive release (Fig.4), but this was less than that seen in Transwell co-culture, suggesting that the inhibitory factor is labile.

### AEC Wounding Attenuates AEC-dependent HLMC Inhibition

**Figure 5:**
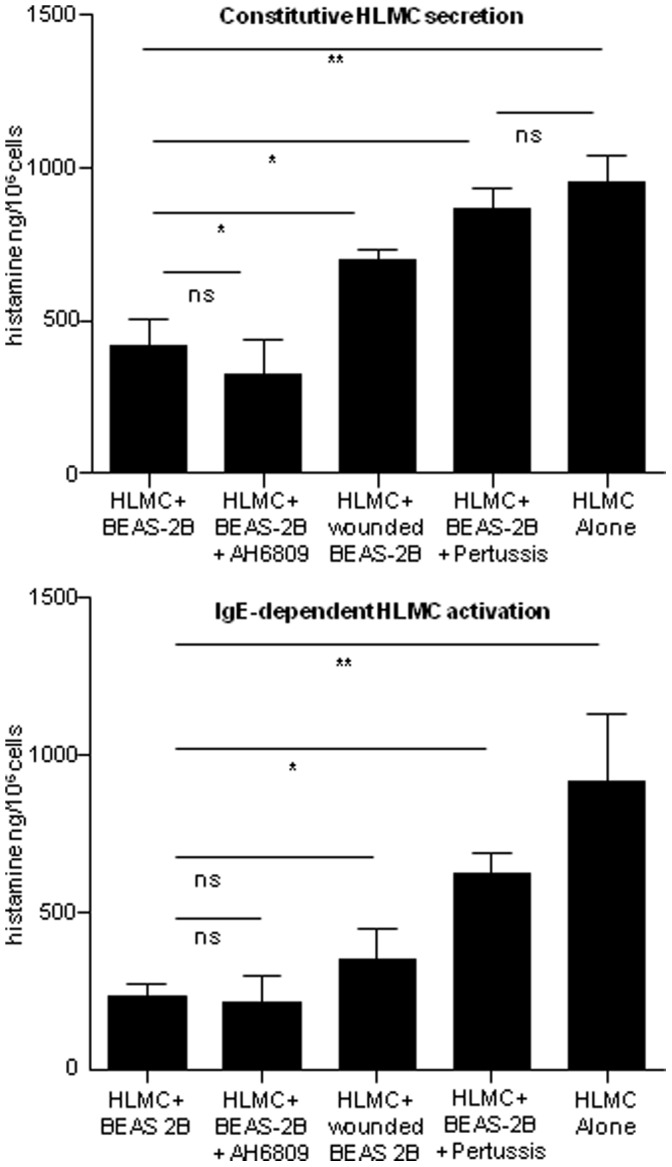
The effects of AH6809, epithelial wounding and pertussis toxin on epithelial-dependent inhibition of HLMC histamine secretion n = 4 HLMC donors, mean ± SEM ng/10^6^ cells. (p<0.05*, p<0.01**, P<0.001***, ns = not significant).

To investigate further the mechanism(s) surrounding the inhibitory effect of AEC on HLMC, firstly we wounded a BEAS-2B Transwell monolayer subtly by scraping the monolayer twice with pipette tip to create an X of damaged cells. Interestingly, this manoeuvre markedly reduced the inhibition of the 16 h constitutive histamine release from HLMC (p = 0.03), but had a much lesser effect on IgE-dependent histamine release (p = 0.29) (Fig. 5) (n = 4 HLMC donors). This might be in part because wound healing over the 16 h restored some inhibitory effect that was active when the HLMC were subsequently activated with anti-IgE.

### EP_2_ Receptor Blockade does not Abrogate AEC-dependent HLMC Inhibition

PGE_2_ is a candidate molecule released by AEC which is known to inhibit HLMC IgE-dependent mediator release both in vitro and in vivo [19]; [20], via the Gs-coupled EP_2_ receptor [21]; [22]. However, in our previous work, the cyclo-oxygenase inhibitors indomethacin and naproxen had no effect on AEC-dependent HLMC inhibition. In keeping with those observations, a dual EP1/EP2 receptor blocker (AH6809) was without effect, firmly excluding PGE_2_ as a mechanism (Fig. 5).

### Pertussis Toxin Attenuates AEC-dependent HLMC Inhibition

Several anti-inflammatory lipid mediators such as PGE_2_, and resolving mediators of inflammation such as lipoxins, resolvins, and protectins operate through Gi-coupled receptors [22]. We therefore incubated HLMC with pertussis toxin (500 ng/ml) overnight in the presence of BEAS-2B AEC Transwell monolayers. This resulted in the complete abrogation of the AEC-dependent inhibition of constitutive histamine release and marked attenuation of the AEC-dependent inhibition of IgE-dependent degranulation (n = 4 HLMC donors) (Fig. 5).

### Resolvin D1 and D2 and Lipoxin A_4_ Attenuate HLMC Histamine Release

**Figure 6:**
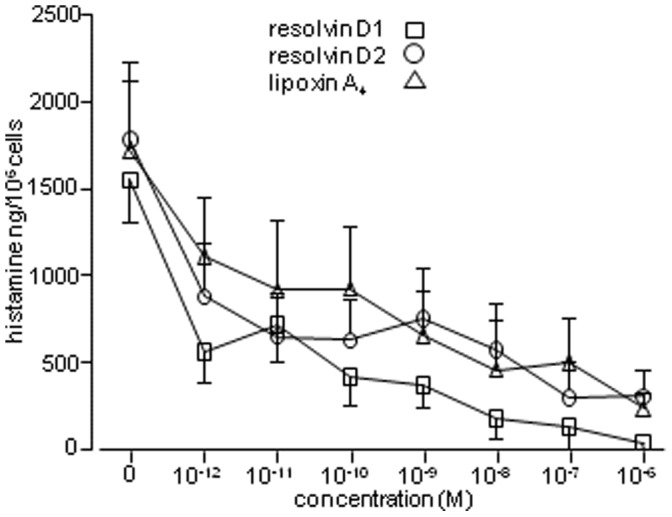
The effects of lipoxin A4, resolvin D1 and resolvin D2 on HLMC IgE-dependent degranulation. Mean ± SEM from 7 HLMC donors. p<0.001 for all mediators (repeated measures ANOVA).

Resolving mediators of inflammation are currently considered to be produced with a delay of several hours following tissue damage, but it is plausible that there is some basal “tone” in this system promoting tissue quiescence. Importantly, these molecules operate through Gi-coupled receptors. Interestingly, preincubation of HLMC with resolvin D1, D2, or lipoxin A_4_ for 10 minutes, produced a marked dose-dependent inhibition of subsequent IgE-dependent HLMC histamine release in the dose range 10^−12^ to 10^−6^ M (Fig. 6). Even at 10^−12^ M, HLMC histamine release was reduced by about 50% compared to vehicle control with each of these mediators (Fig. 6).

### Measurement of Resolving Mediators in Culture Supernatants

A commercial ELISA was used to measure lipoxin A_4_ in the co-culture supernatants. The lower limit of detection was 0.47 pg/ml but no lipoxin A_4_ was detectable. No commercial assays are available for the resolvin family. To assess further whether a related small lipid mediator may be involved, we fractionated AEC supernatants from primary AEC monolayers by removing lipid mediators using reversed phase C^18^ solid phase extraction (SPE)(Strata-X, 33 µm, 85 A°, <10 kDa). Removal of lipid mediators ablated the inhibitory activity present in these AEC supernatants (Fig. 4). As we were able to transfer inhibitory activity in primary AEC monolayer supernatants, we used liquid chromatography (LC)-mass spectrometry (MS) to look for the presence of resolving mediators (lipoxins, resolvins, protectins, maresins) in both primary AEC monoculture supernatants and AEC-HLMC-co-culture supernatants.

**Figure 7:**
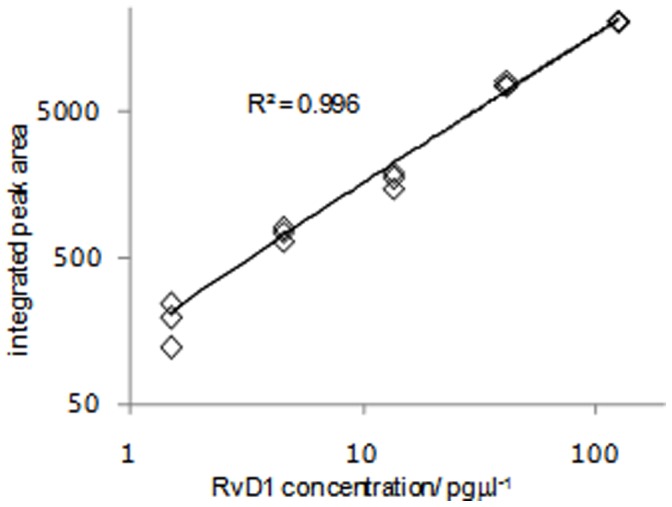
LC-MS calibration curve for triplicate injections of resolvin D1 standard with a linear regression best fit line. Integrated peak area refers to the 375.2 → 141.2 ion pair.

We could accurately and reproducibly measure lipoxin A_4_ and resolvins D1 and D2 with a lower detection limit of 1 pg/µl (2.7 nM/L) for resolvin D1, and 0.5 pg/µl (1.3 nM/L) for resolvin D2 and lipoxin A_4_ (Fig.7). This compares favourably with the detection limit of 11 nM/L for resolvin D1 described by others (23). Although we could reliably detect lipoxin A_4_, resolvin D1 and D2 in spiked AEC media and spiked supernatants, we were unable to identify lipoxin A_4_, resolvin D1 or D2 in AEC/AEC-HLMC co-culture supernatants (n = 12). We could also not detect other resolving mediators including resolvin E1 and E2, protectin D1 and maresin 1 which were screened for using published SRM characteristics as listed in Methods.

## Discussion

We demonstrated previously that BEAS-2B AEC exert a strong suppressive effect on both constitutive and IgE-dependent HLMC degranulation [12]. Suppressive activity in these early experiments was not significant in freeze-thawed culture supernatants. In this study we have extended our work looking at this interaction between HLMC and human AEC, both in the form of the cell line BEAS-2B and in primary AEC ALI cultures and undifferentiated primary monolayers. We have shown that the inhibition of HLMC degranulation by BEAS-2B is also seen with primary human ALI AEC, and that direct cell contact is not required for this effect. Interestingly, this activity can be transferred, although less efficiently, in the supernatant of primary AEC monolayers, suggesting the presence of a soluble mediator(s).

This mediator is not PGE_2_, but signals through a G_0_/Gi protein coupled receptor mechanism as evidenced by the reversal of this inhibition in the presence of pertussis toxin. We suggest that good candidate molecules for this AE-derived suppressive effect are the family of resolving mediators of inflammation which include the lipoxins, resolvins and protectins [23]. Several lines of evidence support involvement of this class of mediators: i) they have already been shown to be important in epithelial cell function and repair [24] and to have a potential role in airway hyperresponsiveness [Bibr pone.0043545-Christie1] [Bibr pone.0043545-Tahan1] [25−27] and asthma [28]; ii) they work through G_0_/Gi protein coupled receptors [29]; iii) their synthesis often requires a contribution from AEC [30]; iv) we have demonstrated for the first time that lipoxin A4, resolvin D_1_ and resolvin D_2_, are potent inhibitors of HLMC degranulation and v) biochemical fractionation indicates that the activity in AEC supernatant is a <10 kDa lipid which would fit the profile of a resolving mediator.

Resolving mediators are generated from arachidonic acid (Lipoxin A4 and B4), docosahexaenoic acid (protectins D1–D4, resolvin D1–D4, maresin 1), and eicosapentaenoic acid (resolvin E1) through biochemical synthesis involving the enzymes 5- and 15-lipoxygenase (5-LOX, 15-LOX) [31]. Several molecules are generated through transcellular synthesis with 15-LOX active in one cell such as an epithelial cell, and 5-LOX active in a second cell such as an inflammatory leukocyte. In addition, aspirin-triggered (AT) forms of these molecules exist (epi-lipoxin A4, AT-resolvins), where acetylated COX-2 generates the initial metabolite which is then modified further by 5-LOX. However, several molecules can be formed endogenously in the absence of aspirin, possibly via a cytochrome P450-dependent pathway [32], and 5-LOX-independent pathways for the production of protectins and maresins also exist [31], with unicellular synthesis evident (reviewed in detail in [31]. Interestingly, human mast cells express both 5-LOX and 15-LOX, providing another potential mechanism for unicellular synthesis [33]; [34].

The resolving mediators we tested exhibited a marked inhibition of HLMC degranulation at concentrations well below the current detection limits of current lipoxin ELISAs (approximately 10^−10^ M) or high performance mass spectrometry techniques (approximately 10^−8^ M [35]. Measuring these in their biologically active range is therefore challenging. We were unable to detect lipoxin A_4_, resolvin D1 or resolvin D2 using LC-MS although spiked standards were detected in culture media, and could not detect Lipoxin A_4_ by ELISA. We could also not detect other resolving mediators (resolvin E1 and E2, protectin D1 and maresin 1) by LC-MS which were screened for using published SRM characteristics. However, such molecules could well be present and exert significant biological activity well below the currently available levels of detection. As well as the characterised resolving mediators mentioned above, there are also potentially hundreds of other arachidonic acid, eicosapentanoic acid and docosahexanoic acid metabolites which might exert similar effects [36].

Three further independent studies support our findings [Bibr pone.0043545-Finotto1] [Bibr pone.0043545-Peden1] [37−39]. Mast cells freshly isolated from human nasal polyp epithelium released only 2% of their histamine following IgE-dependent activation compared to 22% from cells freshly isolated from the polyp stroma. In contrast, both populations of mast cells released ∼50% of their total histamine with calcium ionophore [37]. Hsieh and colleagues demonstrated that primary AEC cultures maintain the survival of human cord blood-derived mast cells, and although not discussed, reduced histamine, PGD_2_ and LTC_4_ release compared to mast cells alone by ∼50% [39]. Furthermore, rat basophilic leukaemia (RBL) cell degranulation was inhibited by a soluble mediator of <3 kDa present in the supernatant of BEAS-2B [38]. Interestingly, RBL cells are able to synthesise lipoxins when fed with appropriate 15-lipoxygenated precursors such as 15-HPETE [40]; [41], indicating that our hypothesis for the involvement of these mediators in our observations is plausible.

In the current work we have investigated the effect of primary AEC derived from both healthy and asthmatic subjects. ALI cultures derived from healthy subjects behaved in a very similar fashion to BEAS-2B, exhibiting a strong suppressive effect on both constitutive and IgE-dependent HLMC histamine release. Interestingly, ALI cultures from asthmatic individuals were less effective at suppressing constitutive HLMC histamine release, but were still very effective at inhibiting IgE-dependent release. This raises the possibility that there is an intrinsic defect in asthmatic AEC which results in loss of the brake on background mast cell activity, which may therefore predispose to the development of atopy and asthma. Alternatively there may be two mediators active in this system, one which targets constitutive mast cell mediator release and which is relatively deficient in asthma and another which targets IgE-dependent release.

The signalling pathways involved in IgE-dependent mast cell degranulation are known in some detail [37]; [39]. However, little is known about the factors influencing constitutive mast cell mediator release. Our data show clearly that this can be modulated. IgE-dependent activation results in the fusion of granules and the formation of large degranulation channels, so-called anaphylactic degranulation [42]. While this appearance can be seen in asthmatic airways, the typical mast cell morphology here and in other disease tissues is that of piecemeal degranulation, where the granule membranes remain intact with variable loss of granule contents [43]. Whether this is an extension of the constitutive release evident in vitro, or yet another release pathway is not known. If the former, then the loss of AEC inhibitory activity in asthmatic airways could explain the morphological appearance of mast cells in asthma, and the chronically elevated concentrations of mast cell-derived mediators identified previously [3]. The impaired inhibition of constitutive HLMC histamine release by asthmatic AEC and the further loss of inhibition following AE wounding, may therefore be a fundamental abnormality which contributes to the development and propagation of asthma.

Epithelial damage in asthma can be profound with almost complete loss of the epithelial layer [13]; [44]. It is noteworthy, therefore that subtle damage to the AEC induced by a pipette tip markedly attenuated the inhibitory effect of AEC on HLMC histamine release. This was most evident when looking at constitutive HLMC histamine release, but also with IgE-dependent release. This suggests that a dynamic process was operative, rather than mechanical loss of AEC being responsible. Identifying the inhibitory mediator(s) and understanding the AEC signalling pathways behind this are important future goals.

In summary we have demonstrated that primary asthmatic AEC are less effective at suppressing the constitutive release of histamine from HLMC than healthy AEC, while both attenuate IgE-dependent degranulation. These suppressive effects are attenuated by AEC wounding, and by disabling G_0/i_-coupled receptors. A soluble lipid mediator(s) is involved, and the family of lipid-derived resolving mediators are candidates, supported by the observation that lipoxin A4, resolvin D1 and resolvin D2 are potent inhibitors of HLMC IgE-dependent activation. These mediators may therefore have potential use as mast cell-targeted therapies. Identifying the inhibitory mediator(s) derived from AEC and understanding the AEC signalling pathways behind its production offers exciting potential for the development of novel approaches to treating asthma.
